# Vasculogenic Chronic Ulcer: Tissue Regeneration with an Innovative Dermal Substitute

**DOI:** 10.3390/jcm8040525

**Published:** 2019-04-17

**Authors:** Barbara De Angelis, Fabrizio Orlandi, Margarida Fernandes Lopes Morais D’Autilio, Chiara Di Segni, Maria Giovanna Scioli, Augusto Orlandi, Valerio Cervelli, Pietro Gentile

**Affiliations:** 1 Department of Surgical Science, Plastic and Reconstructive Surgery Unit, University of Rome Tor Vergata, 00133 Rome, Italy; bdeangelisdoc@gmail.com (B.D.A.); fabrizio.orlandi86@gmail.com (F.O.); mflmdautilio@gmail.com (M.F.L.M.D.); chiara_disegni@virgilio.it (C.D.S.); valeriocervelli@virgilio.it (V.C.); 2 Department of Biomedicine and Prevention, Pathologic Anatomy Institute, University of Rome “Tor Vergata”, 00133 Rome, Italy; scioli@med.uniroma2.it (M.G.S.); orlandi@uniroma2.it (A.O.)

**Keywords:** dermal substitute, Nevelia, chronic ulcers, arterial, venous, collagen

## Abstract

The healing of venous and arterial ulcers is slow, and in some cases, they may not heal at all. This study aims to demonstrate the clinical advantage of Nevelia^®^, an innovative collagen dermal template substitute (DS) in venous and arterial chronic ulcers treatment. 35 patients affected by chronic vascular ulcers with a mean area of 35.1 ± 31.8 cm^2^ were treated with DS followed by autologous dermal epidermal graft (DEG). Follow-up was performed at 7-14-21 and 28 days after DS implant and 7-14-21 and 28 days after DEG. At 28 days after DEG, the mean values of Manchester Scar Scale was of 1.8 ± 0.7 for skin color, 1.6 ± 0.7 for skin contour, 1.7 ± 0.7 for distortion, and 1.7 ± 0.7 for skin texture, whereas skin was matte in 27 patients (77%) and shiny in the remaining eight cases (23%). Histological findings correlate with the clinical result showing a regenerated skin with reactive epidermal hyperplasia and dermal granulation tissue after two weeks (T1), and after three weeks (T2) a re-epithelialization and a formed new tissue architecture analogue to normal skin physiology. These data suggest that Nevelia^®^ could be useful to treat chronic venous and arterial ulcers.

## 1. Introduction

Vasculogenic ulcers (venous, arterial, and/or both) are the most common type of ulcers in the legs. They are chronic and may last from weeks to years [1,2] and show high recurrence rates [3]. Of these ulcers, 50% return within 10 years and 20% of patients experience 10 or more ulceration episodes, and 9.3% develop more than one ulcer in both legs [4]. Ulcers that do not heal within six to eight weeks have the risk of becoming infected and the infection may result in an increase in the ulcer size, delayed healing or amputation [5].

A venous leg ulcer (VLU) results from damage to superficial and/or deep vein valves, impairing venous return, causing increased venous pressure, and compromising the supply of oxygen and tissue growth factors [6,7]. Arterial leg ulcers are associated with the interruption or decrease of blood flow, resulting in tissue ischemia and cell death due to nutrient and oxygen deficits and are often associated with atherosclerosis and diabetes [8]. Mixed leg ulcers are a combination of the chronic venous insufficiency and peripheral arterial occlusive disease [9,10]. Vasculogenic ulcers, are 56–70% of venous origin, 10–20% are arterial, and 9–26% have mixed etiology [11,12]. Approximately 1–3% of the population is affected and the prevalence grows with age [13,14,15]. 

This study treated both arterial and arterial-venous mixed ulcers. Some patients, even after successful re-vascularization do not achieve healing success in the wound healing or their reduction [13].

Moreover vasculogenic ulcer treatment costs are elevated due to recurrent hospitalization, complications, such as edema, and infection [13,14,15]. Venous leg ulcers negatively affect the patient’s quality of life, causing exudate, bad smell, pain, depression, social isolation, personal hygiene difficulties, and limitations in physical mobility, and, in severe cases, amputation [15,16,17]. Arterial and mixed leg ulcer treatment includes revascularization, debridement, systemic therapies (antibiotics, analgesia, pharmacotherapies), patient education and nutrition [18,19]. For VLUs, while compression therapy is the recommended intervention based on guidelines, a high level of evidence and elevation of the lower limb are necessary [20]. Advanced dressings and topical agents (e.g., hydrocolloids, alginates, hydrogels, foams, antimicrobials) are applied to protect the ulcer surface, absorb the exudate, and provide comfort and a humid environment ideal for healing. The healing of VLUs is slow, and in some cases, they may not heal at all [21]. Venous leg ulcers do not progress through the normal physiological phases of healing, but remain in the inflammatory phase: Increased levels of metalloproteinases (proteases) in the inflammatory phase destroy proteins essential for extracellular matrix (ECM) formation [22,23]. Without these proteins, angiogenesis does not occur and cellular adhesion in the ECM is degraded, delaying healing [24].

Extracellular matrix technologies that can be derived from human sources (allografts) (e.g., donated human skin) or animal sources (xenografts) (e.g., porcine, equine, bovine) [25,26] are part of the tissue engineering, with several products available in the market [27,28]. These matrix technologies aim to stimulate matrix components or replace the damaged matrix to optimize wound healing. A-cellular matrix products are processed to remove animal or donor cells, leaving the collagen matrix preferably maintaining the physiological 3D structure [28]. The matrix can provide a tissue scaffold for the patient’s own cells, allowing cellular interaction for migration, proliferation and differentiation, thus promoting re-epithelialization, revascularization and closure [29,30,31].

This study aims to demonstrate that Nevelia^®^, an innovative collagen dermal template substitute, can be used successfully not only in burns and traumatic ulcers [32] but also in vasculogenic chronic ulcers non-responder to conventional treatments.

## 2. Materials and Methods

This retrospective observational case-series study was conducted following the principles outlined in the Declaration of Helsinki and internationally consented ethics in clinical research [33]. A quality assessment was carried out based on the Strengthening the Reporting of Observational studies in Epidemiology (STROBE) checklist [34]. The study protocol, object of an inter-university master’s degree called “Regenerative surgery of loss of substance”, was approved with Rectoral Degree (D.R. n. 1693/2016) of July 12, 2016 and the Ethics on Research Committee of the School of Medicine, “La Sapienza” University, Rome, Italy, and School of Medicine, “Tor Vergata” University, Rome, Italy, with registration number #27696, which is where clinical activities were performed. All patients received detailed oral and written information about the study, including the risks, benefits and alternative therapies, and signed an informed consent form before any study procedures.

### 2.1. Patients

From January 2015 to September 2018, 35 patients affected by vasculogenic chronic ulcers localized on the inferior limbs (17 patients affected by venous ulcers, 11 patients with arterial ulcers and seven patients with mixed arterial/venous ulcers) were treated with the application of collagen dermal substitute template (DS) Nevelia^®^ followed by the autologous dermal-epidermal graft (DEG) (Nevelia^®^ Protocol: DS + DEG) at the Department of Plastic and Reconstructive Surgery, University of Rome, “Tor Vergata”, Italy. 

**Chart 1:**
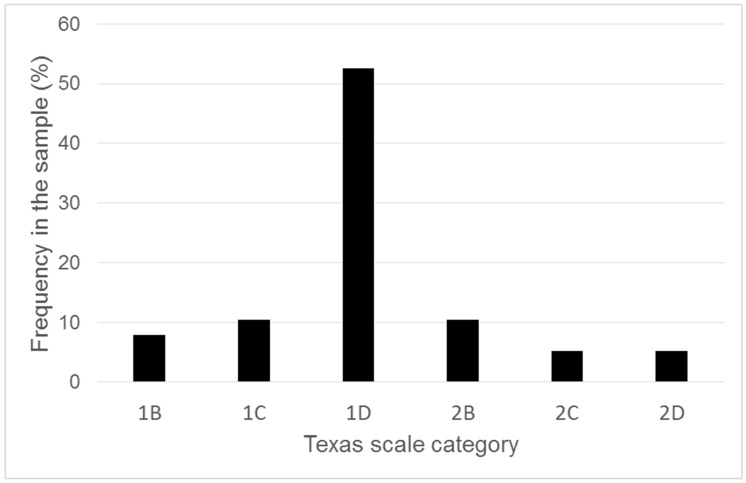
The frequency of each category of Texas scale in the sample of enrolled patients.

Patients mean age was 61.91 ± 18.05, 15 of them were women (42.9%), the mean area of ulcers before the intervention was 35.1 ± 31.8 cm^2^, with a high variability, going from 4 to 180 cm^2^. The authors evaluated the area and dimensions of the ulcers according to the Texas University Wound Classification. Most of the patients were classified with a Texas score of 1D (20 out of 35, 57.1%—green line in Table 1), whereas three were classified as 1B (8.6%—pink line in Table 1), four as 1c (11.4%—light blue line in Table 1), four as 2B (11.4%—blue line in Table 1), two as 2C (5.7%—red line in Table 1), and two as 2D (5.7%—yellow line in Table 1), as shown in Chart 1. In addition, the patient’s characteristics with their different comorbidity, dimensions of the ulcers were also reported in Table 1. Inclusion criteria were arterial, mixed and venous ulcers also with tendons exposures non-responder after eight weeks of conventional treatments. 

### 2.2. Dermal Substitute

Nevelia^®^ Bi-Layer Matrix (Symathese Biomateriaux – Chaponost – France), consists of a porous resorbable matrix of about 2 mm thickness made of stabilized native collagen type I and a silicone sheet of about 2 mm in thickness mechanically reinforced with a polyester (200 μm) fabric. For details see Table 2. 

### 2.3. Clinical and Surgical Protocol 

**Figure 1:**
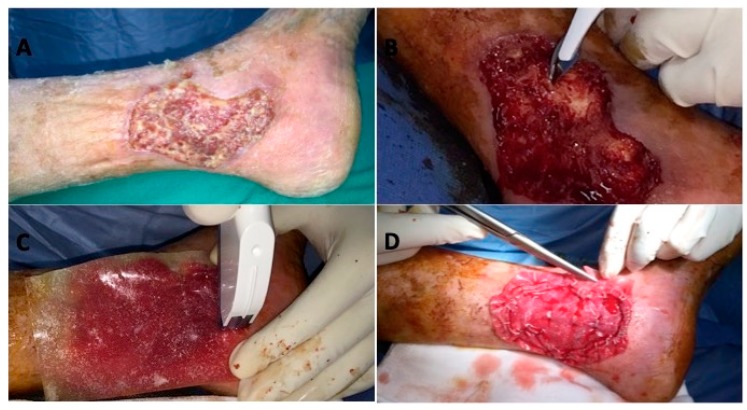
Surgical Protocol. (**A**) Vascular ulcer, pre-operative situation; (**B**) careful debridement; (**C**) implant of dermal substitute; (**D**) remodelling.

**Figure 2:**
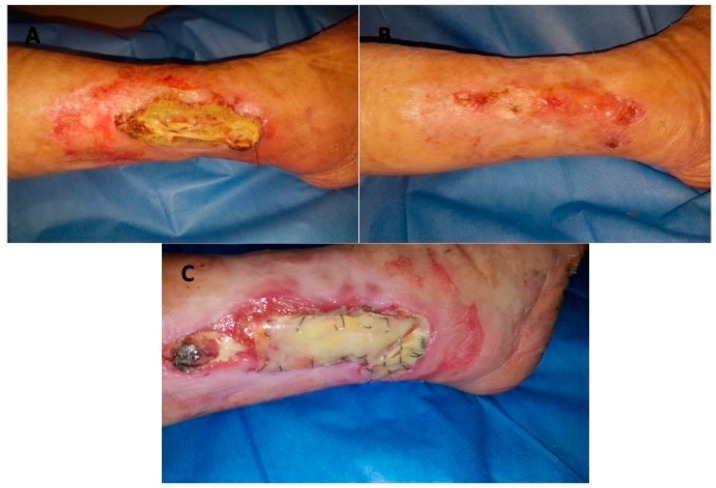
Clinical case. (**A**) Preoperative situation of patient affected by vascular ulcer of left lower limb; (**B**) Postoperative situation showing healing 28 days after DEG; (**C**) Follow-up period during DS application.

Complete asepsis was performed followed by sedation or epidural and/or regional loco anaesthesia. The first surgical step consisted in a careful debridement (Figure 1B) of the injured tissue (Figure 1A) and the implant of dermal substitute (Figure 1C,D). Simple wound dressings were performed, every seven days, during the time interval between the first and second surgery. After 28 days the silicon sheet (Figure 2C) was removed and the autologous skin graft (Dermal Epidermal Graft -DEG) was implanted (Figure 2B). DEG was applied with a dermatome, after the silicon layer removal. Metallic staplers fixed DEG to the wound and sterile gauze was applied over it in order to protect the surgical wound. The bandage was performed.

### 2.4. Clinical Criteria Evaluation During Treatment and Follow-up

The percentage of patients with complete re-epithelialization, were recorded during the observation period to evaluate the effectiveness of the dermal substitute healing. Clinical results were determined through the Manchester Scar Scale (MSS), the Visual Analogue Scale (VAS) was recorded for patient self-estimation though the Rosenberg scale and for pain evaluation. The Manchester Scar Scale is based on five parameters for the scar evaluation: Colour, skin texture, contour, distortion and texture giving a score of one (excellent) to four (poor) for all parameters except skin texture that is represented by score one or two (matte or shiny). Patient self-estimation (Rosenberg scale) was performed in terms of the functional and aesthetic outcome. It consisted in grading results from one to four (1—very disappointed; 2—disappointed; 3—satisfied; 4—very satisfied). Post-operative pain was evaluated with the Visual-Analogical Scale (VAS—Range value: 0–10). Follow–up was performed at 30 days after DS implant and 30 days after DEG. Photographs were taken at each follow-up time. Also, all patients were followed at 1-3-6 and 12 months after healing. The aim of the work was to demonstrate the wound healing 28 days later after the DEG application and perform serial controls during the follow-up to identify the eventual relapse of ulcer.

### 2.5. Histological Evaluation

Incisional punch biopsies (3 mm in diameter) of ulcers were obtained at baseline (T0) and after dermal substitute application at T1 (after two weeks) and T2 (after three weeks). Microscopic evaluation of routinely Haematoxylin & Eosin-stained paraffin [35,36] was performed to verify the healing process and images were acquired by using a digital camera (E600 Eclipse, Nikon, Melville, NY, USA). Moreover, to evaluate the presence of elastic and collagen fibers in the dermis Verhoeff-Van Gieson, staining was also performed.

### 2.6. Immunohistochemical Study 

For immunohistochemistry [37] 22, 4-μm thick serial sections were deparaffinised, rehydrated and, after antigen retrieval and non-specific peroxidise blocking, incubated with mouse monoclonal anti-human CD31 (DakoCytomation, Glostrup, Denmark) and alpha-SMA (DakoCytomation) [38] and images were acquired by using a digital camera.

### 2.7. Statistical Analysis

Data were reported in terms of mean, standard deviation and percentage of total number of cases in the text, whereas in Figures data were summarized and reported in terms of median values and quartiles. This allows depicting an overall meaningful summarization of the recorded data. The prevalence of re-epithelialization percentage was reported using the Kaplan Meier plot. The wilcoxon test was used to compare the scores of VAS given for pain before and after the intervention. The software used for the statistical analysis was the IBM SPSS Statistics software package version 23 (IBM Corp., Armonk, NY, USA).

## 3. Results

### 3.1. Wound Features 

**Chart 2:**
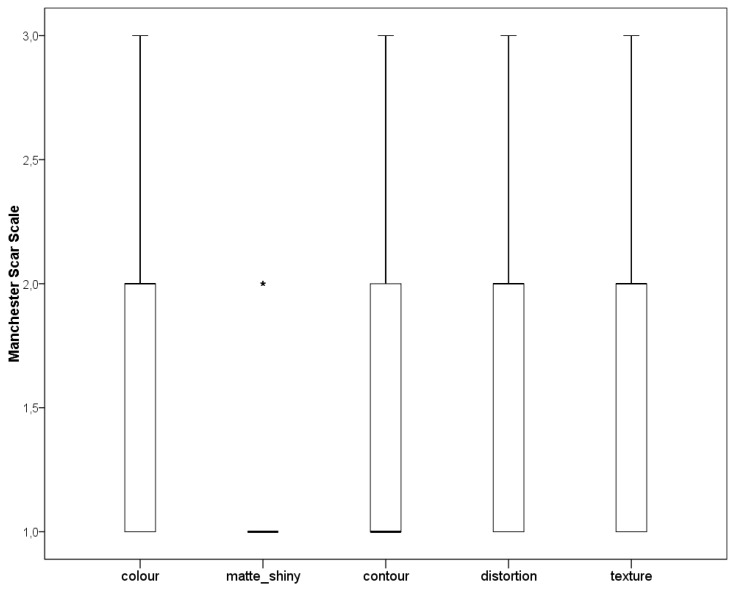
Manchester scar scale (MSS) at 28 days after dermal epidermal graft (DEG). Box whiskers plot reporting median (bold line), first and third quartiles (borders of the boxes) and minimum and fourth quartiles (borders of whiskers). For matte shiny, all the data converged into the line with the exception of one patient, represented by the star and considered as an outlier because out of 1.5 of the inter-quartile range.

**Chart 3:**
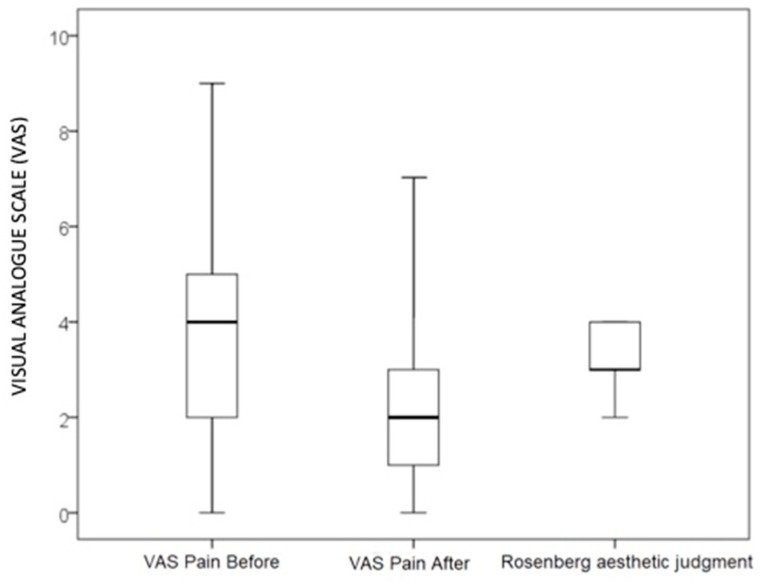
Visual analogue scale (VAS). Box whiskers plot reporting median (bold line), minima (lower line), first and third quartiles (borders of the boxes) and fourth quartiles (borders of whisker) of VAS-score about pain before and after intervention and the aesthetic judgment given using Rosenberg-scale.

**Chart 4:**
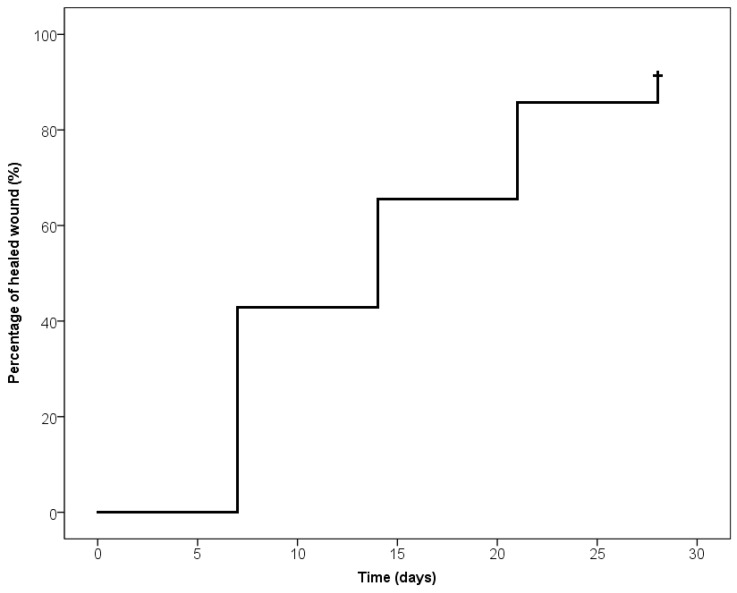
Kaplan-Meier plot. The percentage of completely healed-wound during the observation period.

After 28 days weeks of the DEG implant, the mean values of the Manchester Scar Scale was 1.8 ± 0.7 for skin colour, 1.6 ± 0.7 for skin contour, 1.7 ± 0.7 for distortion, and 1.7 ± 0.7 for skin texture, whereas the skin was matte in 27 patients (77%) and shiny in the remaining eight cases (23%). The median and quartile values of the Manchester Scar Scale are reported in Chart 2. Chart 3 shows the means of VAS-scores that patients reported in terms of pain (1.4 ± 1.6) and the self-estimation of aesthetic result (1.4 ± 0.5) 30 days after the DEG implant. The VAS-score associated to pain was significantly lower after intervention with respect to before the intervention (*P* < 0.01). Chart 4 reports the Kaplan Meier plot for the percentage of completely healed-wounds with complete re-epithelialization during the observation period of 28 days after DEG. 

### 3.2. In Vivo Evaluation

**Figure 3:**
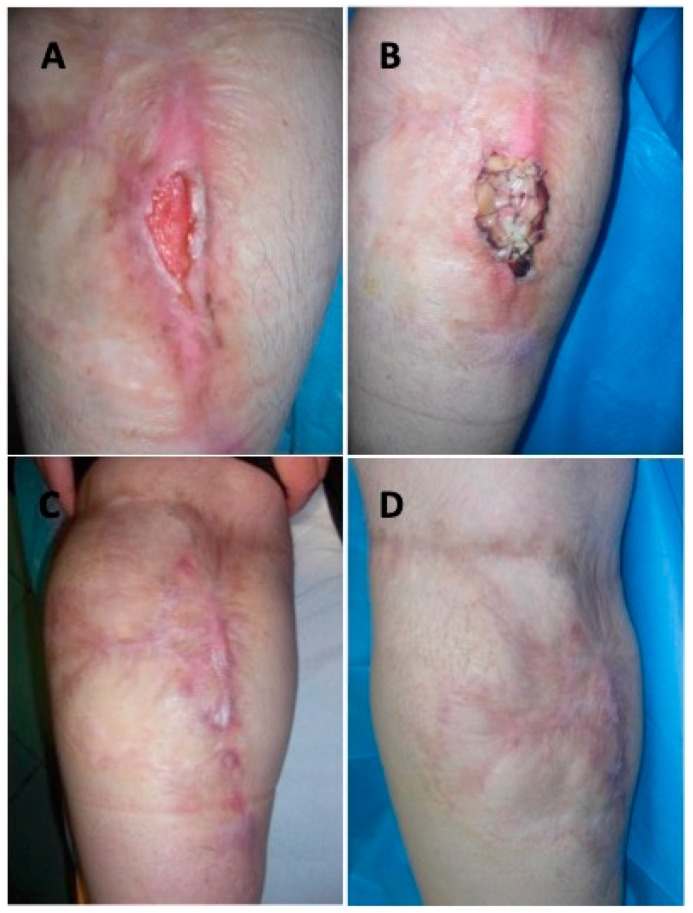
Clinical case. (**A**) Preoperative situation of patient affected by vascular ulcer of the back portion of the calf; (**B**) Follow-up period during DEG application; (**C**) postoperative situation showing healing 28 days after DEG; (**D**) post-operative situation one year after DEG.

Combined material, autologous skin graft and dermal matrix are successfully integrated in the treated area. The new regenerated tissue resulting favourable and comfortable for tissue damage for deficit of tissue. The collagen maturation (neo-angiogenesis) is evaluable through the transparent silicon layer. The change of colour (Figure 2C) indicates that the wound bed is ready for the DEG implant (Figure 3B). 28 days after DEG, 33 patients (94%) were completely healed, of these 17 patients were affected by venous ulcers, nine patients by arterial ulcers and seven by mixed arterial/venous ulcers. In detail, during the follow-up evaluation, seven days after DEG 16 patients (46%) (eight patients with venous ulcers, three with arterial ulcers and five mixed) were completely healed; 14 and 21 days after DEG, respectively eight patients (23%) (five patients with venous ulcers, two with arterial ulcers and one with mixed) and seven patients (20%) (four patients with venous ulcers, two with arterial and one mixed) were completely healed; two patients (6%) with arterial ulcers were completely healed in 28 days. However, two remaining patients (6%) affected by arterial ulcers didn’t heal but the reduction of the wound area already achieved about 80% of the initial area. The clinical result of patients affected by vascular ulcer (Figure 2A, Figure 3A) treated with DS and followed by DEG was reported in Figure 2B, Figure 3C,D.

### 3.3. In Vitro Evaluation

**Figure 4:**
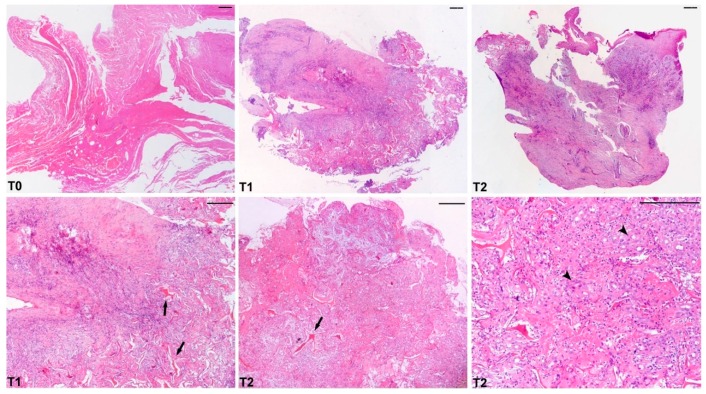
Microscopic aspects of wound healing and neo-angiogenesis after Nevelia^®^ application. Representative microscopic images of Hematoxylin & Eosin-stained paraffin sections of skin biopsies at baseline (T0) showing a typical ulcer with cellular debris. After the dermal substitute application at T1 (after two weeks) and at T2 (after three weeks), skin punch biopsies showed wound healing with re-epithelialization, dermal granulation tissue with collagen deposition and newly formed vessels (arrowheads). The presence of Nevelia^®^ is still evident in dermal tissue at T1 and T2 (arrows). Scale bar = 5 µm.

Representative microphotographs of Haematoxylin & Eosin staining are reported in Figure 4. An evident healing of wounds was already documented after two weeks from the DEG implants (T1) and more markedly after three weeks (T2) compared to T0 with Nevelia^®^ dermal substitute. In particular, T0 biopsy showed cellular debris with no evidence of collagen deposition. At T1, a regenerated skin with reactive epidermal hyperplasia and dermal granulation tissue were observed, along with inflammatory infiltrate and collagen deposition around the dermal substitute (arrows). At T2, re-epithelialization and dermal regeneration were evident with the replacement of Nevelia^®^ substitute with new dermal tissue; interestingly Nevelia^®^ showed regenerative properties also in terms of newly formed vessels (arrowheads).

## 4. Discussion

Vasculogenic ulcer is a very common affection. It is an impaired wound healing due to many factors such as compromised oxygen supply and tissue growth factors reduction. Also, it is susceptible to infections [35,36,37,38]. 

Extracellular matrix technologies stimulate matrix components or replace the damaged matrix in order to accelerate the wound-healing process. Therapeutic angiogenesis, through biological ECM, has proven to be a safe and effective treatment for venous ulcers and mixed arterial/venous ulcers. The Bi-Layer Matrix has shown early striking and long-term effects together with a favourable safety profile, significantly decreasing the risk of amputation [24,39]. Moreover, Nevelia^®^ dermal substitute is safe and easy to use in a sterile operating environment. Dermal skin substitute (DS) is a valuable treatment option, particularly for those difficult patients with chronic ulcers who did not respond to conventional treatments.

In arterial ulcers, the revascularization method by itself, not always guarantee healing and often, is burdened by failure in diabetic patients where the peripheral vascular bed and the microcirculation are strongly compromised.

In some diabetic patients, even after successful under the knee revascularization, trophic lesions do not heal or heal very slowly: It was recently reported by Okazaki et al. [13] that out of 304 re-vascularized patients, both in open (bypass, 170 patients) and with percutaneous transluminal angioplasty (PTA) (194 patients) lesion healed percentage at one, two and three years was respectively 56.3%, 63.4% and 64.0%. In the group of patients with healing lesions, patients treated with PTA, the Wound Healing Time (WHT) was higher (217 days) than patients treated with the surgical bypass (116 days), with an average of 158.2 days.

Shiraki et al. [40] in a retrospective study of 871 consecutive patients with critical limb ischemia (CLI) and revascularization with PTA, of which 734, with trophic lesions and 553 diabetics, showed a wound healing rate of 67% after one year (33% with lesions not healed after ine year). The one-year data of 33% of unhealed lesions was also confirmed by Robinson et al. [41], which publishes an average healing time of 209 days after revascularization.

Moreover, Reed et al. [42,43] in a retrospective study of 179 patients with CLI and tissue loss, demonstrated through the Kaplan-Meyer statistical analysis, that the probability of major adverse events (MALE-major amputation, surgical endarterectomy, or bypass) was higher in patients with trophic lesions that did not heal within four months of revascularization (log-rank *P* < 0.0001) compared to those with healed lesions. Patients with non-healing lesions also had a higher rate of amputation (HR, 9.0, 95% CI, 2.6–31.1; *P* = 0.0004), while patients with lesions healed in three months showed the lowest amputation rate (log-rank, *P* = 0.04). The same authors have also published a retrospective study of 252 patients treated with endovascular CLI procedures and are re-hospitalized after 30 to 180 days for unhealed trophic lesions [43]. From this data it is clear that chronic wounds, both arterial and mixed arterial/venous, represent a strong unmet medical need so far.

Dermal skin substitutes (DS) represent a main group of extracellular matrix products providing ECM replacement in the form of porous three-dimensional dermal templates to stimulate wound healing [24,26,28,39]. They represent a relatively new therapeutic option in chronic wound management [44,45] with a growing evidence base in diabetic and venous ulcers [30,31]. Recently, Tchero et al. [46] in a systematic review with meta-analysis on dermal substitute, conclude that the data showed an overall low failure rates suggesting that these bioengineered skin products provide a suitable support and microenvironment for healing of diabetic foot ulcers but that is a pressing need for more studies. 

DS available on the market (Integra^®^, Nevelia^®^, Matriderm^®^ and Pelnac^®^) showed a similar structure (bilayer of collagen and silicon sheet) but also main differences such as porosity, elasticity, decellularization process, collagen 3D structure, cross-linker type and density, presence or absence of glycosaminoglycans (GAG), stability to temperature, resistance to proteases, are likely to affect multiple wound healing processes including fibroplasia, angiogenesis, cellular migration and finally the quality of the new formed derma [39,45,47,48]. Historically, biomaterials were designed to be inert to minimize the host response. More recently, the emergence of tissue engineering and regenerative medicine has led to the design of biomaterials that interact with the host immune system that can positively interact with the immune system to dictate a favourable macrophage response [49]. Due to an improved understanding of macrophage responses to implanted materials, it is now possible to identify biomaterial design characteristics that dictate the host response and contribute to successful tissue integration. Briefly, the pro-inflammatory (M1) vs. anti-inflammatory (M2) behaviour is typically compared between biomaterials, with the promotion of M2 being suggested as a desired outcome [49,50]. The macrophage polarization into M2 anti-inflammatory state in response to inherent cues provided by biomaterials is playing a key role in angiogenesis and tissue regeneration [50]. So far, the polarization response has been published only for Integra^®^ [51] showing a mixed M1/M2 response. 

Sindrilaru et al. [52,53] have shown that iron overloading of macrophages—as was found to occur in human chronic venous leg ulcers induced a macrophage pro-inflammatory M1 activation via an enhanced TNF-α and hydroxyl radical release, and that this macrophage population perpetuated inflammation and induced a p16INK4a-dependent senescence program in resident fibroblasts, eventually leading to impaired wound healing. For this reason, biomaterials used on chronic venous leg ulcers should preferably elicit an anti-inflammatory M2 prone behaviour. 

In our study, we observed that 33 patients out of 35 were completely healed at 28 days, which means four weeks after the DEG implant and that the two patients remaining showed 80% reduction of the wound area. This preliminary result, although it is an “observational” data, devoid of the comparison to a control group, suggest that the implant of Nevelia^®^ dermal substitute can be used on critical and high-risk patients to heal vascular lesions that were not responding to any other conventional treatment. 

Our data, that more than 90% of patients healed after four weeks from the implant, agrees with data previously reported by Romanelli et al. [54] where the application of a biological ECM (OASIS Matrix) on VLUs and mixed arterial/venous ulcers, resulted in 80% healing, compared to 65% with standard care alone (*P* < 0.05). 

Moreover, regarding the quality of the new formed tissue both clinical MS score, VAS aesthetic -self -estimation score together with iconographic results, showed a positive result. This data is strongly supported from the histological evaluation where an evident healing of wounds was already documented after two weeks from the DEG implants and more markedly after three weeks, suggesting the resumption of the normal reparative physiological response that had remained blocked for eight weeks before the Nevelia^®^ implant, as confirmed from the T0 biopsy that showed cellular debris with no evidence of collagen deposition. After two weeks, (T1) a regenerated skin with reactive epidermal hyperplasia and dermal granulation tissue were observed, along with inflammatory cells infiltrate mainly formed by macrophages, and collagen deposition around dermal substitute. At T2, re-epithelialization and dermal regeneration were more evident and Nevelia^®^ substitute start to be replaced by a new dermal tissue. Interestingly, Nevelia^®^ showed regenerative properties also in terms of newly formed vessels clearly visible in the new formed tissue. Tissue regeneration and angiogenesis are strongly linked [50]: Nevelia^®^ showed a positive histological response when implanted on chronic vascular lesion, as previously observed in post- traumatic lesion [32].

Our data on pain measured through the VAS score suggest that Nevelia^®^ implant can control the ulcers linked pain as reported by Shores et al. [55] where the application of a-cellular matrix on mixed leg ulcers has resulted in a better life quality by providing comfort, pain reduction and lowering time-to-dressing change [55]. 

Based on the experience reported, Nevelia^®^ gave a positive result when applied to vasculogenic ulcers. Surgical technique is simple and the healing time is reasonable. Moreover, as previously reported, DS stimulates both neo-angiogenesis and tissue regeneration by forming new tissue architecture analogue normal skin physiology. This treatment consists in cost reduction and hospitalization, improving the patient’s quality of life. Patients demonstrated better clinical outcome. In these types of patients, it is important to be reminded of the recurrence’s control and prevention. 

The treatment in two steps, DS and SG, warrants skin protection because of its many roles such as haemostatic, reduction of contracture wound, infection, maintenance of skin elasticity and dermal architecture, and scar appearance improvement.

## 5. Conclusions

In conclusion, we obtained satisfied results in our study in terms of safety, versatility, costs and compliance for the patient and surgeon. The treatment with DS Nevelia^®^ is considered an innovative tool in regenerative surgery, a medical-surgical discipline that has been evolving in recent years and that, along with cellular therapy and other innovative biotechnology, is the future of tissue regeneration. More studies with better follow-up (over one year) are necessary to demonstrate the absence of relapse of other vascular ulcers. 

## Figures and Tables

**Table 1:** Patient’s characteristics with the comorbidity, Texas scale grade and dimensions of the ulcers. Abbreviations: Diameter (Ø), Centimeters (cm), Anemia (A), Dyslipidemia (DLP), Diabetes Mellitus (DM), Hemodialysis (HD), Liver Failure (LF), Hypertension (HPT), Heart Failure (HF), Respiratory Failure (RF), Kidney Failure (KF), Vascular Insufficiency (VI), Diabetic Neuropathy (DN), Obesity (OB), Diabetic Retinopathy (DR).

Patients	Age	Gender	Texas	Ø Ulcer T0 (cm)	Comorbidity
**1**	24	M	1B	5 × 4	DM, HPT, DR
**2**	39	F	1B	8 × 4	VI
**3**	45	M	1D	11 × 4	HPT, DLP
**4**	82	M	1C	5 × 3	HPT, HF, HFTUS, VI, P
**5**	77	M	1D	9 × 4	HPT, HF, DM, DLP, DR
**6**	38	F	1D	5 × 3	VI
**7**	59	M	1D	3 × 2	VI
**8**	61	M	1D	4 × 8	DM, DLP, DR
**9**	54	M	1D	2 × 2	DM, HPT, HF, VI, DLP, OB
**10**	67	M	1B	4 × 3	DM, DLP, HF, KFC, DR
**11**	72	M	1D	5 × 10	HF, KFC, DLP, VI, HPT
**12**	74	F	1C	7 × 5	HF, KFC, DLP, HPT
**13**	75	F	1C	2 × 2	DLP, DM
**14**	38	F	1C	5 × 2	KFC, HPT
**15**	33	F	1D	12 × 4	DM, KFC, HF, A, VI, HPT, DR
**16**	77	M	1D	7 × 5	HF, VI, HPT
**17**	60	F	1D	15 × 5	DM, HF, HPT, DR
**18**	41	F	1D	8 × 2	DM, HF, HPT, DR
**19**	66	M	1D	6 × 4	HF, VI
**20**	81	F	1D	8 × 5	VI, HF
**21**	40	M	1D	3 × 2	KFC, HF, DM, A, HPT
**22**	69	F	1D	8 × 5	VI
**23**	54	M	1D	5 × 3	DM, VI,
**24**	72	F	2D	6 × 8	DM, HPT, HF, VI
**25**	44	F	1D	9 × 5	VI
**26**	67	M	2C	3 × 6	DM, HPT, DLP
**27**	78	M	1D	3 × 8	DM, HPT, KFC, DR
**28**	63	M	1D	7 × 8	DM, HPT, DLP, DR
**29**	93	F	1D	5 × 6	DM 1, KFC, DR, A, DN
**30**	87	M	2B	10 × 8	DM, HPT, DLP, KFC, HF
**31**	33	M	2C	7 × 5	DM, HPT, HF, VI, KFC, DLP, DR
**32**	80	F	2D	15 × 12	HF, DM, DR, HPT, KFC, VI
**33**	71	M	2B	6 × 6	VI, OB
**34**	83	F	2B	7 × 8	DM, HPT, KFC, HF, DLP
**35**	70	M	2B	2 × 3	VI, A, HF

**Table 2:** Nevelia^®^ characteristics and clinical standard protocol.

	NEVELIA^®^
**Description**	NEVELIA bi-layer matrix is a sterile matrix consisting of a type I, purified, stabilized, bovine origin collagen to promote dermal regeneration becoming a vascularised tissue that is histologically very close to the normal dermis and a polyester reinforced silicone layer acting as a pseudo-epidermis. The extraction procedure and the freeze-drying process allow the structure of the collagen into a matrix with optimal hydrophily, pore structure and pore size (20–125 μm). Collagen shows a native 3D open cells structure and absence of Glycosaminoglycans (GAG) to improve cells attachments. NEVELIA^®^ can be used in combination with a thin split thickness skin graft to recreate skin resembling normal skin in terms of function and appearance.
**Procedure**	1 Wound excision: NEVELIA^®^ bi-layer matrix must be applied to clean wounds freshly excised under standard operating conditions after excision of all the dead tissue on and around the graft zone. 2 Implantation of NEVELIA^®^ bi-layer matrix: The matrix must be placed in the excised wound precisely to allow an airtight edge-to-edge joint between the healthy skin and the matrix and reduce the risk of bacterial infection. The matrix can be attached with surgical staples or sutures 3 Plantation of thin split thickness skin graft: The silicone layer is removed after 28 days and, at the same time, the thin split thickness skin graft will be applied (ensure that no hematoma, fluid build-up or infection is present). The staples or sutures must remain in place until the thin split thickness skin graft takes place (about three weeks).
**Indication for use**	NEVELIA^®^ is indicated for dermal regeneration in individuals with skin loss, particularly in the following fields: Burns surgery (third and deep second degree burns), Reconstructive plastic surgery, Traumatology, Chronic Ulcers (including leg ulcers and diabetic foot). It is particularly useful for patients who are unable to supply sufficient donor skin for an autograft at the time of excision or to accelerate the healing in high-risk patients.

## Abbreviations

Dermal Substitute	DS
Dermal epidermal graft	DEG
Venous Leg Ulcer	VLU
Visual Analogical Scale	VAS
Manchester Scar Scale	MSS
Anemia	A
Dyslipidemia	DLP
Diabetes Mellitus	DM
Hemodialysis	HD
Liver Failure	LF
Hypertension	HPT
Heart Failure	HF
Respiratory Failure	RF
Kidney Failure	KF
Vascular Insufficiency	VI
Diabetic Neuropathy	DN
Obesity	OB
Diabetic Retinopathy	DR
Glycosaminoglycans	GAG
Percutaneous transluminal angioplasty	PTA
Critical limb ischemia	CLI
